# Effectiveness of rehabilitation training on radiotherapy-related abnormalities of voice function in head and neck cancer patients: A systematic review and meta-analysis

**DOI:** 10.1371/journal.pone.0318577

**Published:** 2025-03-10

**Authors:** Mengqin Zhao, Jiaying Yao, Junhui Wang, Xinyi Shen, Tingting Liu, Limin Pu, Ying Li, Xiaomin Chen

**Affiliations:** 1 Zhejiang Provincial People’s Hospital, Hangzhou, Zhejiang, China; 2 School of Nursing, Zhejiang Chinese Medical University, Hangzhou, Zhejiang, China; 3 Tongxiang Hospital of Traditional Chinese Medicine, Hangzhou, Zhejiang, China; 4 School of Nursing, Hangzhou Normal University, Hangzhou, Zhejiang, China.; Ankara University, TÜRKIYE

## Abstract

**Background:**

Abnormal speech function caused by radiotherapy will affect the normal communication of patients with head and neck cancer (HNC) and even interrupt their social life. Rehabilitation Training is widely used to improve articulatory abnormalities in patients with HNC. However, the effectiveness of these rehabilitation measures in restoring the voice function of HNC patients is still unknown.

**Objective:**

This study aimed to systematically examine the effects of rehabilitation training on radiotherapy-related voice function and quality of life in patients with HNC.

**Methods:**

The databases PubMed, Web of Science, EMBASE, Cochrane Library, CINAHL, CNKI, Wan Fang, and SinoMed were searched for studies published from inception through October 2024. Randomized controlled trials of rehabilitation training to improve voice function abnormalities associated with radiotherapy for HNC were included, and two investigators independently performed the literature review. Meta-analysis was performed using RevMan 5.4 software to determine statistical heterogeneity based on *P*-values and *I*^*2*^ values.

**Results:**

13 randomized controlled trials involving 710 participants were included. Meta-analysis showed that rehabilitation training significantly affected the patients’ maximum phonation time(MD=1.53, 95%CI=[0.83, 2.23], *P*<0.0001, Grade Moderate), smoothed cepstral peak prominence(MD=-0.59, 95%CI=[-0.89, -0.29], *P*=0.0001, Grade Moderate), social communication abilities(MD=-2.60, 95%CI=[-5.14, -0.07], P=0.04, Grade Moderate), and quality of life(MD=8.49, 95%CI=[3.06, 13.92], *P*=0.002, Grade Moderate).

**Conclusions:**

Rehabilitation training is an effective approach for ameliorating abnormal voice functions after radiotherapy for HNC. However, there is no consensus on the optimal frequency, periodicity, and follow-up of interventions for rehabilitation training. More studies are still required to determine the optimal intervention effect for ameliorating speech function abnormalities in patients with HNC after radiotherapy.

## 1. Introduction

Head and Neck Cancer (HNC) is a malignant tumor that occurs in the larynx, oral cavity, nasal cavity, pharynx, and salivary glands, accounting for 10% to 30% of all malignant tumors. It ranks as the sixth most prevalent malignant tumor globally, exerting significant effects on the overall health of the human population [1]. Radiotherapy serves as a significant auxiliary means in the treatment of HNC and constitutes a crucial component of the definitive treatment plans. Currently, it is extensively employed in the treatment of HNC. Nevertheless, it may trigger a series of related complications such as vocal cord scarring, laryngeal edema, glandular atrophy, and fibrosis in patients in the course of radiotherapy [2]. Radiation damage induced by radiotherapy can be categorized into acute and delayed effects, with acute episodes leading to mucositis, edema, necrosis, and epithelial detachment [3]. A fibroblast reaction occurs after tissue detachment, reducing the elasticity and viscosity of normal tissue, which is caused by the delayed effects of radiotherapy. Normal speech and swallowing function requires precise coordination of vocal cords and muscle structures [4], however, the sequelae of tissue fibrosis and neuropathy caused by radiotherapy may lead to speech function problems such as dysarthria, decreased speech articulation, and poor speech quality [5]. One study revealed that 55% of patients with advanced HNC had mild to severe dysphonia after radiotherapy, which persisted for up to 11 years post-treatment [6,7]. The speech impediment will hinder patients’ ability to communicate effectively, thereby diminishing their overall quality of life [8], and even causing the interruption of patients’ social life and bringing serious mental trauma to patients [9]. The influence of functional communication changes on interpersonal relationships and self-awareness of HNC patients is irreversible [10]. Hence, medical professionals must address the issue of speech perception in patients with HNC undergoing radiation and devise optimal rehabilitation strategies to enhance speech functionality [11].

Nevertheless, the current body of research on speech technology is restricted, with the majority of studies primarily concentrating on developing tools and models to assess and forecast speech function [12,13]. Rehabilitation training is a training approach for ameliorating radiotherapy-related speech dysfunction in HNC through voice training, swallowing training, or other functional exercises. Currently, several research has investigated the impact of rehabilitation training on speech problems caused by radiation for HNC. Nevertheless, the findings of these studies have presented contradictory results. Several studies have demonstrated [5] that voice training has a long-lasting impact on voice quality in patients with early-stage vocal cancer. In contrast, the research conducted by Zhang et al [14] revealed that voice training did not have a beneficial impact on patients with laryngeal cancer who had undergone radiation therapy (*P*<0.05). The Research data show that voice training is beneficial in promoting self-perceived communication skills and quality of life in patients undergoing radiotherapy for laryngeal cancer [15]. Moreover, rehabilitation training is designed to ameliorate the abnormal vocal function of patients with laryngeal cancer and facilitate their early return to work, thereby effectively alleviating the medical burden and economic stress on society [16,17].

The psychological and economic burden of HNC patients should not be underestimated [18,22]. Nevertheless, at present, there is considerable controversy regarding the research results of the influence of rehabilitation training on the speech function of HNC patients, and the validity of intervention measures still requires further investigation [19]. Therefore, this study undertook a novel systematic review and meta-analysis, encompassing all pertinent randomized controlled trials, to investigate the impact of rehabilitation training on subjective evaluation of voice function, acoustic analysis, social communication ability, and quality of life. In addition, we conducted a subgroup analysis of the outcomes that exhibited significant heterogeneity in order to investigate the underlying cause of this heterogeneity. The purpose of this study is to furnish medical and nursing personnel with evidence-based rehabilitation interventions to improve the speech dysfunction of HNC patients caused by radiotherapy.

## 2. Methods

This study was conducted by the Preferred Reporting Items for Systematic Review and Meta-analysis (PRISMA) guidelines [20], and the content of the list is shown in S1 Table. The study was registered on the PROSPERO website with registration number CRD42024539332.

### 2.1 Literature search strategy

A total of eight databases, PubMed, Web of Science, Embase, Cochrane Library, CINAHL, CNKI, Wan Fang, and SinoMed were systematically searched by a combination of medical subject terms and keywords. The period frame extended from the inception of the database to October 2024. Search terms included head and neck cancer, head and neck tumors, radiotherapy, radiation therapy, rehabilitation training, voice function, voice rehabilitation, speech rehabilitation, and voice therapy, with results limited to randomized controlled trials. The detailed search strategy for the database is shown in the S2 Table.

### 2.2 Eligibility criteria

This study developed literature inclusion and exclusion criteria based on PICOS principles. The inclusion criteria were as follows (1) Population: adult head and neck cancer patients (≥18 years of age) who have not undergone previous surgical treatment (or have undergone surgical treatment without postoperative pathologic changes in the vocal cords) and who require a definitive radiation regimen. Meanwhile, it is required that the patients have good cognitive function and be capable of cooperating with the therapists to complete the treatment. Ethnicity, treatment type, clinical stage, and pathological stage were not restricted;(2) Intervention: rehabilitation exercises such as speech training, swallowing training or other functional exercises;(3) Control: the control group received routine nursing or other intervention measures (such as placebo, blank);(4) Outcome: subjective evaluation indicators of speech quality (such as voice impairment index, hoarseness assessment) objective evaluation indicators of speech quality (such as voice analysis), social communication ability, quality of life evaluation indicators (QOL); (5) Design: randomized controlled trials.

The exclusion criteria were as follows: (1) Patients with pathologic changes in the vocal cords after undergoing surgical treatment for head and neck cancer;(2) absence of outcome indicators related to voice function; (3) unavailability of full text or relevant data; (4) poor results of literature quality assessment; (5) case reports, conference papers, qualitative studies, reviews, research protocols, and articles from non-randomized controlled trials;(6) duplicate publications.

### 2.3 Study selection and data collection

The imported literature was screened by Endnote X9 literature management software. After the initial weight removal, the remaining literature was independently screened by two researchers based on the eligibility criteria. If there was a disagreement over the screening findings, a third researcher was approached for review.

### 2.4 Data extraction and analysis

The structured data templates are pre-designed, and the relevant data are independently extracted into the templates by two researchers. The details of the template are shown in the S4 Table. The relevant data include (1) general information: first author, publication year, country; (2) subject characteristics: sample size, age; (3) intervention characteristics: intervention content, intervention frequency, duration; (4) evaluation details: evaluation tools, measurement results. The results of the measurement will be extracted after the intervention, and if some of the data are missing, the researcher will contact the original author of the article by email. If the article only provides a 95% confidence interval (CI), the data will be converted to standard deviation (SD) according to the following formula: 95% CI = X±Z(_α/2_)S_X_. If the article only provides standard error (SE), the data will be converted to standard deviation according to the following formula: SD =SE $\sqrt{\text{n}}$ . If there is a study that cannot be meta-analyzed, the study will be included in the systematic evaluation.

### 2.5 Risk of bias assessment

Two researchers used the Cochrane risk bias tool [21] to evaluate the quality of the included randomized controlled trials, each of which was selected as “low bias risk”, “high bias risk” and “unclear”. When more than three areas included in the article were judged to be high risk, then the overall bias risk of the study was high. Any disagreements were resolved by consulting a third reviewer.

### 2.6 Data synthesis strategy

The extracted data were analyzed using RevMan 5.4 software and sensitivity analysis was performed by Stata 17 software. Statistical heterogeneity was determined according to *P* value and *I*^*2*^ value. If *P* > 0.1 and *I*^*2*^ < 50%, the heterogeneity among studies is low, so the fixed effect model is used for analysis; if *P* ≤ 0.1 and *I*^*2*^ ≥ 50%, heterogeneity among studies is high, random effect model is used for analysis, and then subgroup analysis is carried out according to the possible sources of heterogeneity (intervention frequency, intervention cycle, follow-up time). A meta-analysis was conducted using a significance level of α = 0.05, where *P* < 0.05 was regarded to be statistically significant. The outcome indicators included in this study are continuous variables, so when different voice quality assessment tools were chosen between studies, standardized mean difference (SMD) was chosen as the effect size indicator for calculation. The evaluation tools used in some studies represent better results with higher scores (high priority scale), while those used in some studies represent better results with lower scores (low priority scale). Therefore, the effects need to be separated and combined if necessary. Finally, the relevant evidence was evaluated using the GRADEpro GDT software, and the quality of the evidence was categorized as high, medium, low, and very low.

## 3. Results

### 3.1 Search results

**Fig 1:**
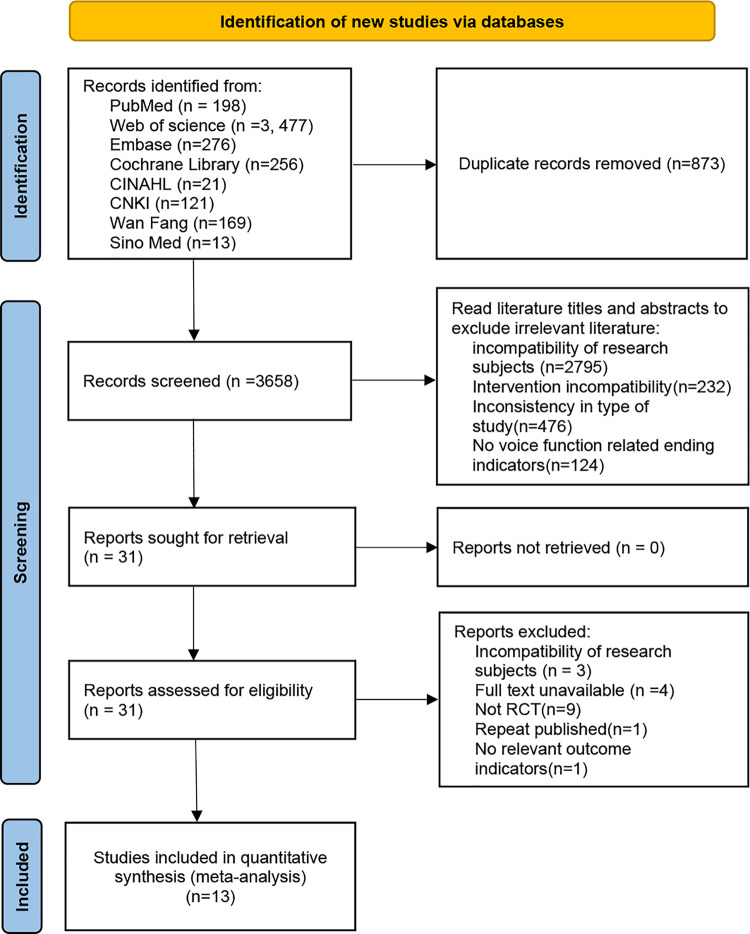
Flow diagram for study selection according to PRISMA.

The details of the PRISMA flow chart for searching and filtering are shown in Fig 1. Our search of 8 databases yielded a total of 4531 records. After duplicated records were eliminated, 3658 studies were identified. The remaining 31 articles are excluded after reading the title and abstract of the article, and 18 articles (Incompatibility of research subjects [n = 3], Full text unavailable[n=4], Not RCT[n = 9], Repeat published[n = 1], No relevant outcome indicators[n = 1]) are excluded through full-text reading. Finally, 13 papers were included for systematic evaluation and meta-analysis. The specific reasons for elimination are presented in S5 Table.

### 3.2 Characteristics of included studies

The specific characteristics included in the study are shown in Table 1. This study included 13 randomized controlled trials [15,16,23–33] with a total of 710 subjects. The sample sizes of the subjects ranged from 10 to 74. Participants were all adults, ranging in age from 50.00 ±11.00 to 69 ±5.34 years old. Related studies on speech function have been carried out in 5 countries. Eight studies [15,16,23–25,28,29,31] were conducted in Sweden, 2 in China [32,33], 1 in the United States [27], 1 in India [30], 1 in Italy [26]. In 9 studies [15,16,23–27,29,31], only laryngeal cancer patients were recruited, while in the other 4 studies [28,30,32,33], nasopharyngeal carcinoma, glottis cancer, hypopharyngeal carcinoma, tonsil cancer, tongue cancer, and other HNC were recruited. Seven studies [16,23,25–27,30,31] were conducted with the combination of voice training and cognitive intervention, 1 study [28] combined head-up exercise and swallowing training, 1 study [32] combined voice training and swallowing training, and 1 study [33] combined neuromuscular electrical stimulation therapy and head-up movement. Three studies [15,24,29] focused only on voice training. The number of rehabilitation training interventions ranged from 4 to 24, and the duration of the intervention ranged from 25 to 60 minutes. All the control groups received routine care, placebo, or blank treatment.

**Table 1:** Characteristics of the included studies.

Author (Year)/Country	Cancer Types	Sample size (Intervention/Control)	Age (Mean ± SD), Y	Intervention Group	Control group	Frequency and duration	Outcome measures/instrumentation	Voice outcome Mean ± (SE or SD) After intervention
Tuomi et al,23 2014, Sweden	laryngeal cancer	I: 33 C: 36	I: 66.0±12.7 C: 64.0±9.9	①Voice training: Practice diaphragm breathing, coordinate breathing and vocal production, and control pitch changes. ②Cognitive intervention: Voice physiology education and voice nursing	Cognitive intervention: voice physiology education and voice nursing	Twice a week in weeks 1-2, once a week in weeks 3-6, and once every two weeks in weeks 7-8, for a total of 10 times.	Acoustic voice analyses, S-SECEL	S-SECEL I:9.6±7.3/C:12.0±8.3 Jitter I: 1.34±1.90/C: 1.43±2.23 Shimmer I: 0.63±0.52/C: 0.66±0.52 NHR I: 17.2±6.9/C: 15.4±6.2 F0 I: 106.8±19.0/C: 107.3±23.6 MPT I: 14.9±9.2/C: 13.5±13.4
Karlsson et al,24 2015, Sweden	laryngeal cancer	I: 37 C: 37	I: 65.0±12.4 C: 62.6±9.7	Voice training: a voice rehabilitation course consisting of relaxation, breathing, pronunciation posture, and vocal exercises	Cognitive Intervention: Provide methods and handouts on voice care	Once a week for 30 minutes for 10 weeks	EORTC QLQ-H&N35, S-SECEL	S-SECEL I: 3.8±2.2/C: 4.5±2.7 EORTC QLQ-H&N35 I: 76.4±18.8/C: 68.9±16.3
Bergström et al,25 2016, Sweden	laryngeal cancer	I: 30 C: 31	I: 65±11.25 C: 62±10.25	①Voice training: a voice rehabilitation course composed of breathing, relaxation, pronunciation posture, and vocal exercises. ②Cognitive intervention: Oral Hygiene Counseling	Cognitive intervention: Oral Hygiene Counseling	Twice a week in weeks 1-2, once a week in weeks 3-6, and once every two weeks in weeks 7-8, for a total of 10 times.	GRBAS	GRBAS: I: 1.87±0.63/C: 1.74±0.68
Law et al,33 2017, Hongkong China	Nasopharyngeal carcinoma	I: 29 C:28	I: 50±11 C: 54±13	①Neuromuscular electrical stimulation: use electrodes to stimulate corresponding muscle groups on both sides of the thyroid cartilage, above hyoid bone and thyroid notch ②Head and neck relaxation exercise	①Swallowing exercises: Shaker’s exercises, Masako exercises, hard swallowing exercises, Mendelsohn exercises ②Head and neck relaxation exercise	2-3 times a week, 60 minutes each time, completed within 4-6 weeks	VHI	VHI I: 18.72±14.94/C: 24.55±26.77
Karlsson et al,15 2017, Sweden	laryngeal cancer	I: 33 C: 32	I: 64.5±12.8 C: 62.1±10.2	Pronunciation training: a voice rehabilitation course consisting of breathing, relaxation, pronunciation posture, and vocal exercises	Cognitive Intervention: Provide methods and handouts on voice care	Once a week for 30 minutes for 10 weeks	EORTC QLQ-H&N35, S-SECEL, Acoustic voice analyses, GRBAS	S-SECEL I: 3.7±2.2/C: 4.4±2.56 Jitter I: 1.19±1.08/C:1.08±1.16 Shimmer I: 0.56±0.44/C: 0.50±0.34 NHR I: 16.9±7.3/C: 18.0±5.8 F0 I: 106.0±18.8/C: 109.3±24.2 MPT I: 14.9±9.6/C: 13.7±12.7 S-SECEL I: 16.8±16.5/C: 21.1±15.3 EORTC QLQ-H&N35 I: 78.9±21.0/C: 68.0±16.4
Tuomi et al,16 2017, Sweden	laryngeal cancer	I: 19 C: 23	I: 64.7±11.8 C: 62.7±8.5	Voice training: a voice rehabilitation course consisting of relaxation, breathing, pronunciation posture, and pronunciation exercises	Maintain daily life and undergo sound screening	Twice a week in weeks 1-2, once a week in weeks 3-6, and once every two weeks in weeks 7-8, for a total of 10 times.	Acoustic voice analyses, S-SECEL, EORTC QLQ- H&N35	S-SECEL: I: 4.4±1.8/C: 4.7±2.6 EORTC QLQ-H&N35 I: 72.1±23.0/C: 65.7±17.1
Mantia et al,26 2018, Italy	laryngeal cancer	I: 10 C: 9	I: 68.4±5.21 C: 64.1±7.18	①Voice training: consists of four exercises including warm-up, stretching, contraction, and resistance exercises ②Cognitive Intervention: Provide methods and handouts on voice care	Cognitive Intervention: Provide methods and handouts on voice care	Twice a day for 6 weeks	VHI, GRBAS, EORTC QLQ-H&N35, Acoustic voice analyses, Aerodynamic assessment	VHI I: 30.22±8.19/C: 25.43±9.47 GRBAS I: 6.62±0.88/C: 8.12±0.74 Jitter I: 0.68±0.34/C: 1.66±0.48 Shimmer I: 2.15±0.99/C: 2.04±1.54 MPT I: 19.73±3.85/C: 17.67±3.62
Angadi et al,27 2020, USA	laryngeal cancer	I: 6 C: 4	I: 57.5±14.2 C: 69±5.34	①Voice training: speech nursing courses are provided for patients by speech pathologists to guide patients’ vocal function exercises. ②Cognitive Intervention: Oral Hygiene Counseling	Cognitive Intervention: Oral Hygiene Counseling	Once a day for 6 weeks	VHI, Acoustic voice analyses, Aerodynamic assessment	VHI I: 32.50±23.02/C: 19.75±16.87 MPT I: 20.15±8.7/C: 16.04±2.56
Eriksson et al,28 2020, Sweden	Tonsil carcinoma, laryngeal cancer, tongue cancer, hypopharyngeal carcinoma	I: 24 C: 26	I: 63.6±9 C: 63.4±7	①Swallowing exercises: Instructing patients in specific body and head movements during swallowing, giving advice on effective eating and drinking ②Head-up exercises: Instruct the patient to perform head-up exercises in the supine position. Sustained head raising (isometric) for 60 seconds, repeated 3 times with 1 minute rest in between, then 30 consecutive repetitions of head raising (isometric)	Swallowing exercises: Instructing patients in specific body and head movements during swallowing, giving advice on effective eating and drinking	3 times a day for 8 weeks	GRBAS, VHI	VHI I: 14.3±15.3/C: 13.9±20.6
Millgard et al,29 2020, Sweden	laryngeal cancer	I: 37 C: 37 H: 25	I: 65.2±12.3 C: 62.9±10.0 H: 63±9.4	Voice training: breathing, relaxation, vocal exercises, participants are asked to do vocal exercises at home	Maintain daily life and undergo sound screening	Once a week for 30 minutes for 10 weeks	Acoustic voice analyses, GRBAS	CPPS I: 3.79±0.90 C: 3.52±1.18
Sreenivas et al,30 2021, India	Oropharyngeal cancer, salivary gland cancer	I: 9 C: 11	-	①Voice training: Speech-language pathologists offer basic exercises in relaxation, posture, breathing, and resonant voice therapy ②Cognitive intervention: a way to provide voice care	Cognitive intervention: a way to provide voice care	Twice a day for 6 months	Acoustic voice analyses, GRBAS, VHI	Jitter I: 0.84±0.30/C: 2.17±0.81 Shimmer I: 2.93±0.75/C: 4.29±0.71 NHR I: 0.12±0.03/C:0.28±0.07 F0 I: 161.29±17.73/C:125.12±12.45
Karlsson et al,31 2022, Sweden	laryngeal cancer	I: 37 C: 37 C: 62.6±9.7	I: 65.0±12.4	Voice training: language pathologists perform speech rehabilitation in a structured way. Courses include breathing, relaxation, and vocal exercises	Cognitive intervention: a way to provide voice care	Once a week for 10 weeks	S-SECEL, Acoustic voice analyses, GRBAS	S-SECEL I: 4.2±2.8/C: 3.8±2.7 Jitter I: 0.9±0.7/C: 0.9±0.7 Shimmer I: 0.4±0.2/C: 0.5±0.3 NHR I: 19.2±5.7/C: 17.8±5.1 MPT I: 14.5±8.3/C: 13.0±10.8
Liu et al,32 2024, China	hypopharyngeal carcinoma, laryngeal cancer, other	I: 35	I: 57.77±9.73	①ABCLOVE speech training: including counseling, activation exercises, breathing exercises, throat exercises, oral resonance exercises, vocal cord function exercises, and lifestyle changes	Swallowing training: including the movement of mouth, lips, tongue, and chin, as well as the movement of throat and neck muscles	ABCLOVE voice training: once a day for 15-20 minutes for 5 months	Acoustic voice analyses, VHI	MPTI: 10.98±1.75/C: 9.49±1.41
		C: 35	C: 58.43±12.69	②Swallowing training: including the movement of mouth, lips, tongue, and chin, as well as the movement of throat and neck muscles		Swallowing training: once a day for 10-15 minutes for 5 months		VHII: 5.23±3.34/C: 5.63±3.60

Abbreviations: VHI, Voice Handicap Index; S-SECEL, Swedish Self-Evaluation of Communication Experience After Laryngeal Cancer; EORTC QLQ-H&N35, The European Organisation for Research and Treatment of Cancer Quality-of-Life; GRBAS, the Grade-Roughness-Breathiness-Asthenia-Strain scale; CPPS, smoothed cepstral peak prominence.

### 3.3 Bias risk of inclusion in the study

**Fig 2:**
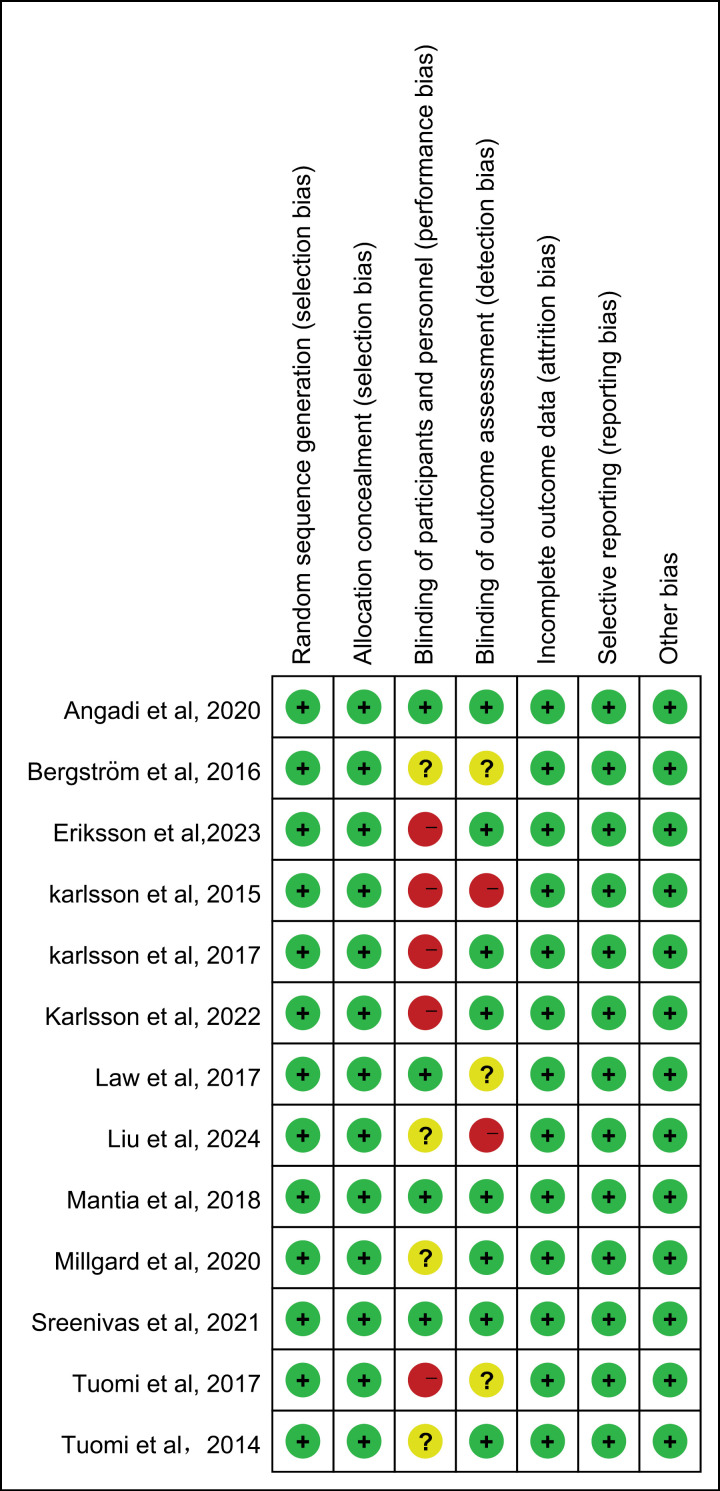
Methodological quality of the trials. (a) The risk of bias graph. (b) The risk of bias summary.

In this study, the Cochrane risk bias tool was used to rate the quality of the included study, and the results are shown in Fig 2a and 2b). In terms of random sequence generation, all studies provide clear and high-quality random sequence generation schemes. Four studies [15,16,24,31] did not blind study participants and interveners, 1 study [28] did not blind interveners, and 4 studies [23,25,29,32] did not explicitly provide information about blinding, so it was impossible to judge whether the above studies applied the blind method to the subjects and interveners. Two studies [24,32] did not blind outcome raters, and information on whether outcome raters were blinded was inadequately described in 3 studies [16,25,33]. All studies were at low risk of bias in terms of lost visit bias, reporting bias, and other biases. In the meta-analysis of the studies, the overall quality of the evidence will be ranked from low to medium, as detailed in the S3 Table. The specific details for quality evaluation are presented in the S6 Table.

### 3.4 Effectiveness of the intervention

#### 3.4.1 Subjective evaluation of voice functions.

**Fig 3:**
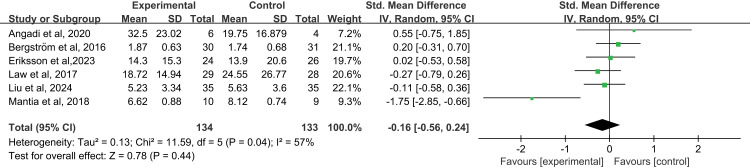
The forest plots subjective evaluation of voice functions.

Six studies [25–28,32,33] subjectively evaluated voice functioning from both the patient and outcome assessor perspectives, with the assessment tools being low-priority scales and effect sizes combined using the SMD, and the analyses showed no statistically significant differences between the test and control groups in subjective ratings of voice functioning (SMD=-0.16, 95%CI=[-0.56, 0.24], *P*=0.44; Fig 3), with larger results in the test of heterogeneity (*I*^*2*^=57%, *P*=0.04). Subgroup analyses showed that the intervention period and follow-up time were not sources of heterogeneity, and the differences were not statistically significant (S7 Fig). Finally, we performed a results sensitivity analysis, which showed a relatively stable result, with a combined effect size of -0.17(95%CI= [-0.59, 0.25]; S8 Fig).

#### 3.4.2 Acoustical analysis.

**Fig 4:**
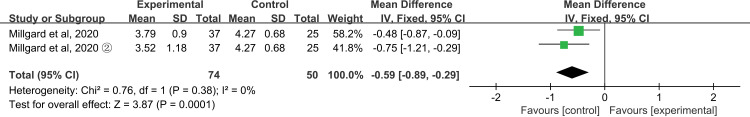
The forest plot of CPPS.

3.4.2.1 **CPPS:** Two studies [29] assessed the smoothed cepstral peak prominence (CPPS) of three groups of patients, which is one of the robust acoustic indicators for evaluating dysphonia [34]. The evaluation tools were the same high-priority scales, and MD was used to combine the effects. Compared with the control group, the rehabilitation training measures had a beneficial impact on the CPPS, and there was a statistical difference between the two groups (MD=-0.59, 95%CI= [-0.89, -0.29], *P*=0.0001; Fig 4), and the heterogeneity test result was low (*I*^*2*^=0%, *P*=0.38).

**Fig 5:**
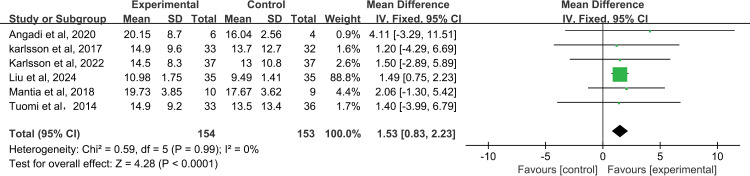
The forest plot of MPT.

3.4.2.2 **MPT:** Six studies [15,23,26,27,31,32] evaluated the maximum phonation time(MPT) of 307 patients with a high priority scale and combined the effects with MD. Compared with the control group, rehabilitation training had a positive effect on the longest vocal time of patients, and there was a significant difference between the two groups(MD=1.53, 95%CI=[0.83, 2.23], *P*<0.0001; Fig 5), and the heterogeneity test result was low(*I*^*2*^=0%, *P*=0.99). The result of the sensitivity analysis was 0.36(95%CI= [0.05, 0.67]; S8 Fig).

3.4.2.3 **Jitter:** In 5 studies [15,23,26,30,31], the voice jitter of 242 patients was evaluated with a low priority scale, and the effects were combined with MD. The results showed that there was no statistical difference in the effect of rehabilitation training on the voice jitter of the patients (MD=-0.47, 95%CI= [-1.06, 0.12], *P*=0.12; S1 Fig) (S1 Fig in S7 Appendix). Given the significant outcomes of the heterogeneity test (*I*^*2*^=86%, *P*<0.0001), this study conducted further analysis based on the predetermined subgroup. In terms of intervention frequency, the frequency of pronunciation training more than 2 times per week has a more significant effect on improving pronunciation jitter (MD=-1.08, 95%CI= [-1.40, -0.76], *P*<0.00001; S5 Fig and the heterogeneity *I*^*2*^ of both groups is less than 50%, indicating that the intervention frequency may be the heterogeneous source of Jitter. In terms of the intervention cycle and follow-up times, the results showed high heterogeneity *I*^*2*^ in both subgroups and the differences were not statistically different (S5 Fig). We conducted a sensitivity analysis of the results, which revealed that the total impact was -0.63(95%CI= [-1.33, 0.06]; S8 Fig).

3.4.2.4 **Shimmer:** In 5 studies [15,23,26,30,31], the voice flicker of 242 patients was evaluated with a low priority scale, and the effects were combined with MD. The results showed that there was no statistical difference in the impact of rehabilitation training on the voice flicker of the patients (MD=-0.12, 95%CI= [-0.36, 0.11], *P*=0.29; S2 Fig). The result of the heterogeneity test is high (*I*^*2*^=71%, *P*=0.008), This study makes further analysis according to the pre-planned subgroup. Subgroup analysis showed no statistically significant differences in frequency of intervention, duration of intervention, and duration of follow-up, and they were not sources of heterogeneity (S6 Fig). Finally, we performed a sensitivity analysis of the results, which showed more stability, with a combined effect size of -0.24(95%CI= [-0.69, 0.20]; S8 Fig).

3.4.2.5 **NHR:** Four studies [15,23,30,31] assessed the noise-to-harmonic ratio (NHR) of 223 patients, which is designed to assess the ratio of harmonic to noise components of speech and is an objective indicator for assessing pathological voice [35]. The assessment tools were high-priority scales, and the effect sizes were combined using the MD. The findings indicated that rehabilitation had a beneficial impact on the harmonic noise ratio of the patients, without any statistical variation (MD=-0.00, 95%CI= [-0.68, 0.68], *P*=0.99; S3 Fig). The test for low heterogeneity (*I*^*2*^=12%, *P*=0.33), we conducted a sensitivity analysis of the results, which revealed that the cumulative impact was -0.27(95%CI= [-0.98, 0.43]; S8 Fig).

3.4.2.6 **F0:** Three studies [15,23,30] assessed the fundamental frequency of the voice in 149 patients, an indicator of the frequency of vocal fold vibration during vocalization, which determines whether a person’s voice sounds bass or treble.[23] The assessment tools for the studies were all low-priority scales and effect sizes were combined using the MD. It was found that there was no statistically significant difference in the fundamental frequency of the patient’s voice by rehabilitation (MD=9.92, 95%CI= [-10.94, 30.78], *P*=0.35; S4 Fig). The forest plot showed a high degree of heterogeneity (*I*^*2*^=89%, *P*<0.0001), and further analysis based on pre-planned subgroups could not be done due to the small amount of literature. We therefore performed a sensitivity analysis of the results, which showed a more stable result, with a combined effect size range of 0.46(95%CI= [-0.45, 1.39]; S8 Fig).

#### 3.4.3 Social communication abilities.

**Fig 6:**
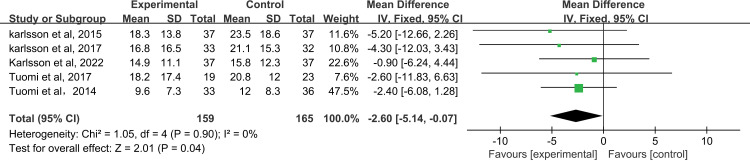
The forest plot of social communication abilities.

The social communication abilities of 324 patients were evaluated in 5 studies. [15,16,23,24,31] The evaluation tools were all low-priority scales, and MD was used to combine the effects. The results showed that rehabilitation training had a positive effect on patients’ social ability, and there was a statistical difference between the two groups (MD=-2.60, 95%CI= [-5.14, -0.07], *P*=0.04; Fig 6), and the heterogeneity test result was low (*I*^*2*^=0%, *P*=0.90). Sensitivity analysis results show more stability-0.23(95%CI= [-0.45, -0.01]; S8 Fig).

#### 3.4.4 Quality of life.

**Fig 7:**
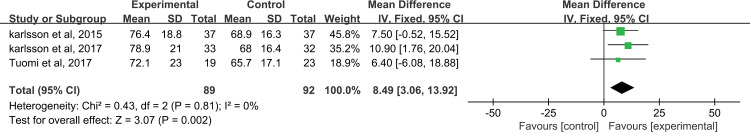
The forest plot of quality of life.

Three studies [15,16,24] assessed the quality of life level of 181 patients using the EORTC QLQ-H&N35 scale, the assessment tool was a high-priority scale, and the effect sizes were combined using MD, and it was found that rehabilitation training had a positive impact on the quality of life level of the patients, with a statistically significant difference between the two groups (MD=8.49, 95%CI=[3.06, 13.92], *P*=0.002; Fig 7), with a low test of heterogeneity (*I*^*2*^=0%, *P*=0.81). Sensitivity analysis results show more stability0.46(95%CI= [0.16, 0.75]; S8 Fig).

### 3.5 Publication bias analysis

Because the outcome index of this study is included in no more than 10 articles, and the efficiency of the funnel chart test is relatively low, publication bias analysis is not carried out.

## 4. Discussion

Systematic review and meta-analysis collected data from 13 randomized controlled trials with a total of 710 participants to evaluate the effectiveness of rehabilitation training on radiotherapy-related speech abnormalities in patients with HNC. There is heterogeneity in the included studies, which may be related to the subtle differences in the implementation of different interventions by Speech-Language Pathologist (SLP). Therefore, we conducted a subgroup analysis of studies with high heterogeneity to further explore the causes of high heterogeneity. In addition, we also evaluate the robustness of each outcome index through sensitivity analysis, and the results are stable and reliable. At the same time, we proved the effectiveness of rehabilitation training in patients’ MPT, CPPS, social ability, and quality of life, and affirmed the value of rehabilitation training in improving speech function in patients with HNC. Therefore, we encourage medical staff to implement speech rehabilitation training programs for patients with HNC speech dysfunction.

### 4.1 Subjective evaluation of voice functions

The subjective evaluation of speech function serves as a straightforward and intuitive approach to comprehend the influence of abnormal speech functions on the physiological, social, and psychological functions of patients. Nevertheless, the study results indicated that there was no statistically significant difference in the subjective evaluation of speech function between the test group and the control group (P=0.44; Fig 3.), which was consistent with the previous meta-analysis [17]. Analyzing the reasons for this, we found that most of the patients suffering from abnormal voice function were also accompanied by dysphagia [36]. Symptomatic experiences from dysphagia can affect patients’ assessment of voice function, which leads to an underestimation of the scores of the subjective evaluations. Furthermore, we can find that vocal training alone has no obvious effect on improving patients’ speech quality, [25–27] while in the study of voice training combined with oral health counseling will get more positive reports on the subjective evaluation of speech quality. It has been shown that poor lifestyle habits (such as smoking) and oral care practices can increase voice burden [37] and that appropriate voice therapy and smoking cessation can help patients better improve their voice function. Consequently, in the design of future studies, an intervention scheme that combines voice training and oral health consultation might better enhance the effect of rehabilitation training in improving speech function. A prospective study [38] showed that during the 6 years of rehabilitation training, patients had fewer functional swallowing and sound problems, confirming the potential of rehabilitation training in improving patients’ language function. Although the improvement in the subjective evaluation of speech function was not statistically significant between the groups, it was observed that the scores of speech function in the experimental group were significantly improved after the intervention. In addition, rehabilitation training can encourage patients to further understand voice nursing strategies, which are closely associated with the improvement of the quality of life related to speech function. Thus, we should not overlook the positive effect of rehabilitation training on enhancing speech function.

### 4.2 Acoustical analysis

Acoustic analysis was performed by taking a 2-second speech sample of the patient’s vowel “a” and scoring the patient’s voice for CPPS, Jitter, Shimmer, NHR, F0, and MPT using an acoustic analysis device. Meta-analysis showed that patients with HNC who received rehabilitation training could achieve better results in terms of MPT and CPPS. However, this study is the first time to use CPPS to evaluate the speech function of patients with HNC, we should carefully interpret the results, which also provides a new perspective for future research.

There is no significant difference in the meta-analysis results of Jitter, Shimmer, NHR, and F0. The measurement and analysis of sound are subject to factors such as recording equipment, analysis software, and microphone distance. Hence, when conducting a comprehensive assessment of the results, we are unable to completely eradicate the impact of these objective differences on the final outcomes. A study [39] concluded that rehabilitative exercise can alleviate the fibrosis of vocal cord tissue caused by radiotherapy, although the difference between the groups was not statistically significant in terms of acoustic analysis parameters, the researchers found that there was no deterioration in acoustic parameters in the test group within one year after radiotherapy, while malignant changes occurred in the control group.Hence, we can’t deny the possibility that rehabilitative training can alleviate the burden of the patient’s voice through measures such as laryngeal relaxation and vocal fold phonation. Due to the high heterogeneity of the study, we performed subgroup analyses of some of the measurements. The results of the study showed that a frequency of intervention greater than 2 times per week was able to produce an effect size in terms of improved phonological function, with a heterogeneity *I*^*2*^ of less than 50% in both groups and that the frequency of interventions may be a source of heterogeneity in Jitter. Future studies with larger samples are needed to explore the effects of different intervention frequencies, intervention periods, and follow-up times in improving voice function in patients with HNC.

### 4.3 Social communication abilities

Social communication skills reflect a patient’s ability to communicate and interact in a specific social context, and the level of a patient’s voice functioning is critical to their social communication. We found that rehabilitation training has a positive effect on social communication ability after intervention (MD=-2.60, *P*=0.04), which suggests that in the process of rehabilitation training, language pathologists or nurses can pay attention to patients’ communication needs and provide patients with more communication skills or communication aids, such as gestures, pictures, and technological aids to help patients overcome communication problems caused by speech disorders [40].

### 4.4 Quality of life

Quality of life serves as a crucial metric for assessing the enhancement of patients’ voice function through rehabilitation training, representing the physical, mental, and social well-being of the patients. From the results of the meta-analysis, there was a significant improvement in the quality of life of the patients after the rehabilitation training intervention (MD=8.49; *P*=0.002), which suggests that the level of patients’ phonological functioning is closely related to the overall quality of life, and this result is in line with the results of previous experiments [41]. Burns et al [42] concluded that HNC patients who receive timely, accurate, and sustained voice-based rehabilitation can maximize the improvement of their quality of life. The ability of telemedicine-based care to provide HNC patients with accurate and continuous rehabilitation training is a boon for improving the quality of life of HNC patients who live in remote areas and have high financial burdens [43]. However, digital medical intervention measures for HNC are relatively scarce, and the number of mobile medical technologies capable of providing rehabilitation training for voice functions is extremely limited [44]. Consequently, in the future research designs, medical staff should attach greater significance to offering services such as language rehabilitation training, oral care consultation, and speech function evaluation for patients via mobile medical platforms, in order to ameliorate the decline in quality of life caused by speech function impairments.

## 5. Limitations

There are some limitations to this study, so the following points need to be taken into account when analyzing the results and planning future research designs: Due to the small number of randomized controlled trials of radiotherapy-related voice function in HNC, a range of HNC types covering nasopharyngeal, laryngeal, and tonsillar cancers were included in this study. The inability to make a more detailed distinction between cancer types due to the limited amount of literature is a major limitation of this study. In addition, radiation dose and radiation region are important factors affecting the degree of speech function impairment. In different randomized controlled trials, even if the same rehabilitation method is used, the feedback results of study patients will be affected by differences in radiation dose and treatment area. Eventually, some studies were rated as moderate bias risk and may have methodological defects (unclear blindness process, inadequate description of blindness by outcome evaluators, etc.). The main reason for the large heterogeneity of the individual pooled outcome indicators may be the diversity of rehabilitation training interventions. Considering the limitations mentioned above, the research results of this review should be interpreted carefully.

## 6. Implications for future practice

In future practical research, we need more research to determine the safety and clinical benefits of rehabilitation training for patients with HNC. In addition, healthcare professionals may consider telemedicine as a way to increase patient satisfaction and participation in rehabilitation training, while simultaneously alleviating the economic strain and healthcare expenses for patients. However, due to the small number of randomized controlled trials included in the study, the optimal intervention frequency, period, and follow-up time of rehabilitation training affecting patients’ voice function cannot be derived, so further studies with larger samples and high quality need to be carried out in the future study design to investigate the optimal intervention effect size for improving the radiotherapy-related language and voice function abnormalities in patients with HNC. Therefore, further studies with larger samples and higher quality should be conducted to investigate the optimal intervention effect size for improving radiotherapy-related abnormalities of speech function in HNC patients.

## 7. Conclusion

Rehabilitation training can improve the social communication ability and quality of life of patients with radiotherapy-related speech dysfunction of HNC and has a significant effect on acoustic analysis parameters such as MPT and CPPS. Through the subgroup analysis, we found that the intervention frequency of more than 2 times per week may be more effective for Jitter. These results are not consistent with the results of previous systematic reviews but can be verified in included randomized controlled trials. The analysis of the results of the subjective evaluation of phonological functioning did not yield significant effect sizes, which may be related to the nuances of speech-language pathologists in the implementation of the different interventions as well as the small sample sizes of the included studies. In addition, from the results of the pre-and post-control scores of each study group, we cannot completely deny the enthusiasm for rehabilitation training in speech function.

## Supporting information

S1 Table PRISMA 2020 checklist. (DOCX)

S2 Table Literature search strategy. (DOCX)

S3 Table Summary of findings. (DOCX)

S4 Table Detailed data. (XLSX)

S5 Table List of excluded studies. (DOCX)

S6 Table Methodological quality of the trials. (DOCX)

S7 Appendix Appendix. (DOCX)
