# Supplementary material for: Effectiveness of rehabilitation training on radiotherapy-related abnormalities of voice function in head and neck cancer patients: A systematic review and meta-analysis
Source: PLoS One. 2025 Mar 10;20(3):e0318577. doi: 10.1371/journal.pone.0318577 (PMC11892882; doi:10.1371/journal.pone.0318577)
Supplement: S2 Table — (DOCX) [file pone.0318577.s002.docx]

| **S1 Table2. Literature Search Strategy** | | | |
| --- | --- | --- | --- |
| **Database** | **Search** | **Query** | **Items Found** |
| Pubmed | #1 | ((((((((((head and neck neoplasms[MeSH Terms]) OR (nasopharyngeal carcinoma[MeSH Terms])) OR (oropharyngeal neoplasms[MeSH Terms])) OR (laryngeal neoplasms[MeSH Terms])) OR (Mouth Neoplasms[MeSH Terms])) OR (Head[Title/Abstract] AND neck cancer[Title/Abstract])) OR (head neoplasm*[Title/Abstract])) OR (neck neoplasm*[Title/Abstract])) OR (head cancer*[Title/Abstract])) OR (neck cancer*[Title/Abstract])) OR (Oral Neoplasm[Title/Abstract]) | 376，547 |
|  | #2 | (((chemoradiation[Title/Abstract]) OR (radiation treatment[Title/Abstract])) OR (Radiotherapy[MeSH Terms])) OR (chemoradiotherapy[MeSH Terms]) | 229，757 |
|  | #3 | (((((((((((((((voice quality[MeSH Terms]) OR (speech therapy[MeSH Terms])) OR (voice[MeSH Terms])) OR (acoustic[MeSH Terms])) OR (dysphonia[MeSH Terms])) OR (aphonia[MeSH Terms])) OR (rehabilitation training [Title/Abstract])) OR (Voice function[Title/Abstract])) OR (voice therapy[Title/Abstract])) OR (voice rehabilitation[Title/Abstract])) OR (speech rehabilitation[Title/Abstract])) OR (vocal function[Title/Abstract])) OR (articulatory function[Title/Abstract])) OR (DYVA[Title/Abstract])) OR (GRBAS[Title/Abstract]) | 69，986 |
|  | #1 #2 AND #3 | ((((((((((((head and neck neoplasms[MeSH Terms]) OR (nasopharyngeal carcinoma[MeSH Terms])) OR (oropharyngeal neoplasms[MeSH Terms])) OR (laryngeal neoplasms[MeSH Terms])) OR (Mouth Neoplasms[MeSH Terms])) OR (Head[Title/Abstract] AND neck cancer[Title/Abstract])) OR (head neoplasm*[Title/Abstract])) OR (neck neoplasm*[Title/Abstract])) OR (head cancer*[Title/Abstract])) OR (neck cancer*[Title/Abstract])) OR (Oral Neoplasm[Title/Abstract])) AND ((((chemoradiation[Title/Abstract]) OR (radiation treatment[Title/Abstract])) OR (Radiotherapy[MeSH Terms])) OR (chemoradiotherapy[MeSH Terms]))) AND (((((((((((((((voice quality[MeSH Terms]) OR (speech therapy[MeSH Terms])) OR (voice[MeSH Terms])) OR (acoustic[MeSH Terms])) OR (dysphonia[MeSH Terms])) OR (aphonia[MeSH Terms])) OR (rehabilitation training [Title/Abstract])) OR (Voice function[Title/Abstract])) OR (voice therapy[Title/Abstract])) OR (voice rehabilitation[Title/Abstract])) OR (speech rehabilitation[Title/Abstract])) OR (vocal function[Title/Abstract])) OR (articulatory function[Title/Abstract])) OR (DYVA[Title/Abstract])) OR (GRBAS[Title/Abstract]) | 198 |
| **Database** | **Search** | **Query** | **Items Found** |
| Web of Science | #1 | Head and neck cancer OR head and neck neoplasms OR head neoplasm* OR neck neoplasm* OR head cancer* OR neck cancer* OR nasopharyngeal carcinoma OR oropharyngeal neoplasm OR cervicofacial cancer OR laryngeal neoplasms OR Mouth Neoplasms OR Oral Neoplasm | 649，854 |
|  | #2 | Radiotherapy OR chemoradiation OR chemoradiotherapy OR radiation treatment* | 1，541，723 |
|  | #3 | rehabilitation training OR Voice function OR voice therapy OR voice rehabilitation OR voice quality OR speech rehabilitation OR speech therapy OR vocal function OR articulatory function OR voice OR acoustic OR dysphonia OR aphonia OR DYVA OR GRBAS | 1,995，476 |
|  | #4 | #1 AND #2 AND #3 | 3，477 |
| **Database** | **Search** | **Query** | **Items Found** |
| Embase | #1 | Head and neck cancer OR head and neck neoplasms OR head neoplasm* OR neck neoplasm* OR head cancer* OR neck cancer* OR nasopharyngeal carcinoma OR oropharyngeal neoplasm OR cervicofacial cancer OR laryngeal neoplasms OR Mouth Neoplasms OR Oral Neoplasm | 83，992 |
|  | #2 | Radiotherapy OR chemoradiation OR chemoradiotherapy OR radiation treatment* | 413，756 |
|  | #3 | rehabilitation training OR Voice function OR voice therapy OR voice rehabilitation OR voice quality OR speech rehabilitation OR speech therapy OR vocal function OR articulatory function OR voice OR acoustic OR dysphonia OR aphonia OR DYVA OR GRBAS | 159，152 |
|  | #4 | #1 AND #2 AND #3 | 276 |
| **Database** | **Search** | **Query** | **Items Found** |
| Cochrane Library | #1 | Head and neck cancer OR head and neck neoplasms OR head neoplasm* OR neck neoplasm* OR head cancer* OR neck cancer* OR nasopharyngeal carcinoma OR oropharyngeal neoplasm OR cervicofacial cancer OR laryngeal neoplasms OR Mouth Neoplasms OR Oral Neoplasm | 27，383 |
|  | #2 | Radiotherapy OR chemoradiation OR chemoradiotherapy OR radiation treatment* | 54，704 |
|  | #3 | rehabilitation training OR Voice function OR voice therapy OR voice rehabilitation OR voice quality OR speech rehabilitation OR speech therapy OR vocal function OR articulatory function OR voice OR acoustic OR dysphonia OR aphonia OR DYVA OR GRBAS | 39，290 |
|  | #4 | #1 AND #2 AND #3 | 256 |
| **Database** | **Search** | **Query** | **Items Found** |
| CINAHL | #1 | Head and neck cancer OR head and neck neoplasms OR head neoplasm* OR neck neoplasm* OR head cancer* OR neck cancer* OR nasopharyngeal carcinoma OR oropharyngeal neoplasm OR cervicofacial cancer OR laryngeal neoplasms OR Mouth Neoplasms OR Oral Neoplasm | 14，032 |
|  | #2 | Radiotherapy OR chemoradiation OR chemoradiotherapy OR radiation treatment* | 59，128 |
|  | #3 | rehabilitation training OR Voice function OR voice therapy OR voice rehabilitation OR voice quality OR speech rehabilitation OR speech therapy OR vocal function OR articulatory function OR voice OR acoustic OR dysphonia OR aphonia OR DYVA OR GRBAS | 30，562 |
|  | #4 | #1 AND #2 AND #3 | 21 |
| **Database** | **Search** | **Query** | **Items Found** |
| CNKI | #1 | 头颈癌 + 头颈肿瘤 + 头颈部肿瘤 + 头颈部恶性肿瘤 + 鼻咽癌 + 鼻咽肿瘤 + 鼻咽部癌 + 舌癌 + 喉癌 + 口腔癌 | 63，590 |
|  | #2 | 放疗 + 放射治疗 | 123，867 |
|  | #3 | 语音功能 + 康复训练 + 言语康复 + 语音训练 + 嗓音评估 + 语音分析 | 5，698 |
|  | #4 | #1 AND #2 AND #3 | 121 |
| **Database** | **Search** | **Query** | **Items Found** |
| WanFang | #1 | 头颈癌 OR 头颈肿瘤 OR 头颈部肿瘤 OR 头颈部恶性肿瘤 OR 鼻咽癌 OR 鼻咽肿瘤 OR 鼻咽部癌 OR 舌癌 OR 喉癌 OR 口腔癌 | 82，240 |
|  | #2 | 放疗 OR 放射治疗 | 164，875 |
|  | #3 | 语音功能 OR 康复训练 OR 言语康复 OR 语音训练 OR 嗓音评估 OR 语音分析 | 152，449 |
|  | #4 | #1 AND #2 AND #3 | 169 |
| **Database** | **Search** | **Query** | **Items Found** |
| SinoMed | #1 | 头颈癌 OR 头颈肿瘤 OR 头颈部肿瘤[MeSH] OR 头颈部恶性肿瘤 OR 鼻咽癌[MeSH] OR 鼻咽肿瘤[MeSH] OR 鼻咽部癌 OR 舌肿瘤[MeSH] OR 喉肿瘤[MeSH] OR 口腔肿瘤[MeSH] | 259，246 |
|  | #2 | 放疗 OR 放射治疗 OR 放射疗法 | 147，570 |
|  | #3 | 语音功能 OR 康复训练 OR 言语康复 OR 语音训练[Mesh] OR 嗓音评估 OR 语音分析 | 185，566 |
|  | #4 | #1 AND #2 AND #3 | 13 |
