# Supplementary material for: Effectiveness of rehabilitation training on radiotherapy-related abnormalities of voice function in head and neck cancer patients: A systematic review and meta-analysis
Source: PLoS One. 2025 Mar 10;20(3):e0318577. doi: 10.1371/journal.pone.0318577 (PMC11892882; doi:10.1371/journal.pone.0318577)
Supplement: S3 Table — (DOCX) [file pone.0318577.s003.docx]

**S2 Table. Summary of Findings for Rehabilitation Training Versus the Control**

| **Certainty assessment** | | | | | | | **№ of patients** | | **Effect** | | **Certainty** | **Importance** |
| --- | --- | --- | --- | --- | --- | --- | --- | --- | --- | --- | --- | --- |
| **№ of studies** | **Study design** | **Risk of bias** | **Inconsistency** | **Indirectness** | **Imprecision** | **Other considerations** | **Intervention** | **Control** | **Relative (95% CI)** | **Absolute (95% CI)** |  |  |
| **Subjective evaluation of voice functions** | | | | | | | | | | | | |
| 6 | randomized trials | serious^a^ | serious^b^ | not serious | serious^c^ | none | 134 | 133 | - | SMD **0.11 SD lower** (-0.35 lower to 0.14 higher) | ⨁◯◯◯ Very low | IMPORTANT |
| **CPPS** | | | | | | | | | | | | |
| 2 | randomised trials | not serious | not serious | not serious | serious^c^ | none | 74 | 50 | - | MD **-0.59 lower** (-0.89 lower to -0.29 lower) | ⨁⨁⨁◯ Moderate | CRITICAL |
| **MPT** | | | | | | | | | | | | |
| 6 | randomized trials | not serious | serious^b^ | not serious | not serious | none | 154 | 153 | - | MD **1.53 higher** (0.83 higher to 2.23 higher) | ⨁⨁⨁◯ Moderate | CRITICAL |
| **Jitter** | | | | | | | | | | | | |
| 5 | randomised trials | serious^a^ | serious^b,d^ | not serious | not serious | none | 120 | 122 | - | MD **0.47 lower** (-1.06 lower to 0.12 higher) | ⨁⨁◯◯ Low | IMPORTANT |
| **Shimmer** | | | | | | | | | | | | |
| 5 | randomised trials | serious^a^ | serious^b,d^ | not serious | not serious | none | 120 | 122 | - | MD **0.12 lower** (-0.36 lower to 0.11 higher) | ⨁⨁◯◯ Low | NOT IMPORTANT |
| **HNR** | | | | | | | | | | | | |
| 4 | randomised trials | serious^a^ | serious^b^ | not serious | not serious | none | 110 | 113 | - | MD **0.00 lower** (-0.68 lower to 0.68 higher) | ⨁⨁◯◯ Low | NOT IMPORTANT |
| **F0** | | | | | | | | | | | | |
| 3 | randomised trials | serious^a^ | serious^b^ | not serious | not serious | none | 73 | 76 | - | MD **9.92 higher** (-10.94 lower to 30.78 higher) | ⨁⨁◯◯ Low | NOT IMPORTANT |
| **Scial communication abilities** | | | | | | | | | | | | |
| 5 | randomised trials | not serious | not serious | not serious | serious^c^ | none | 159 | 165 | - | MD **2.6 lower** (5.14 lower to 0.07 lower) | ⨁⨁⨁◯ Moderate | CRITICAL |
| **Quality of Life** | | | | | | | | | | | | |
| 3 | randomised trials | not serious | not serious | not serious | serious^c^ | none | 89 | 92 | - | MD **8.49 higher** (3.06 higher to 13.92 higher) | ⨁⨁⨁◯ Moderate | CRITICAL |

Abbreviations: CI, confidence interval; MD, mean difference; SMD, standardised mean differenc

a. Unclear risk of bias in multiple domains (Random sequence generation is unclear; no blinding implemented).

b. Inconsistencies in results across studies

c. Relatively low number of incidents observed

d. Quality was rated down for inconsistency because *I^2^* > 50%.
