# Supplementary material for: Effectiveness of rehabilitation training on radiotherapy-related abnormalities of voice function in head and neck cancer patients: A systematic review and meta-analysis
Source: PLoS One. 2025 Mar 10;20(3):e0318577. doi: 10.1371/journal.pone.0318577 (PMC11892882; doi:10.1371/journal.pone.0318577)
Supplement: S5 Table — (DOCX) [file pone.0318577.s005.docx]

**S5 Table. List of excluded studies**

| **Reasons for exclusion** | **Number of studies** | **List of excluded articles** |  |
| --- | --- | --- | --- |
| Incompatibility of research subjects | 3 | 1.Ouyoung LM, Swanson MS, Villegas BC, Damodar D, Kokot N, Sinha UK. ABCLOVE: Voice therapy outcomes for patients with head and neck cancer. Head Neck. 2016;38 Suppl 1:E1810-1813. doi:10.1002/hed.24322 | |
|  |  | 2. Cd van G, Im V de L, Ja L, Dj K, Hf M. Long-term efficacy of voice therapy in patients with voice problems after treatment of early glottic cancer. Journal of voice : official journal of the Voice Foundation. 2012;26(3). doi:10.1016/j.jvoice.2011.06.002 | |
|  |  | 3. van Gogh CDL, Verdonck-de Leeuw IM, Boon-Kamma BA, et al. The efficacy of voice therapy in patients after treatment for early glottic carcinoma. Cancer. 2006;106(1):95-105. doi:10.1002/cncr.21578 | |
| Full text unavailable | 4 | 1.Vocal Rehabilitation after Radiotherapy for Laryngeal Cancer—Pilot Study - Lisa Tuomi, Eva Björkner, 2012. Accessed October 10, 2024. https://journals.sagepub.com/doi/abs/10.1177/0194599812451438a8 | |
|  |  | 2.M A, F B, A R. [Phonetic results in patients irradiated for carcinoma of the vocal cords]. Acta otorhinolaryngologica Italica : organo ufficiale della Societa italiana di otorinolaringologia e chirurgia cervico-facciale. 1987;7(2). Accessed October 10, 2024. https://pubmed.ncbi.nlm.nih.gov/3618210/ | |
|  |  | 3.ChiCTR2300072130. Application effect of voice training combined with swallowing function training in radiotherapy for head and neck cancer. https://trialsearch-who-int/Trial2.aspx?TrialID=ChiCTR2300072130. Published online July 31, 2023. doi:10.1002/central/CN-02583852 | |
|  |  | 4.Timmermans AJ, Krap M, Hilgers FJM, van den Brekel MWM. [Speech rehabilitation following a total laryngectomy]. Ned Tijdschr Tandheelkd. 2012;119(7-8):357-361. doi:10.5177/ntvt.2012.07/08.12132 | |
| Not RCT | 9 | 1. S R, E F, Gc B, Jwg R. The role of the speech and language therapist in the rehabilitation of speech, swallowing, voice and trismus in people diagnosed with head and neck cancer. British dental journal. 2022;233(9). doi:10.1038/s41415-022-5145-2 | |
|  |  | 2. Aggarwal VV, Waghmare CM, Lolage SN, Pawar HJ, Ravichandran M, Bhanu A. Subjective and perceptive assessment of speech/voice and swallowing function before and after radiation therapy in patients of head-and-neck squamous cell cancer. J Cancer Res Ther. 2023;19(Supplement):S0. doi:10.4103/jcrt.jcrt_621_21 | |
|  |  | 3. Sarin V, Chatterjee A. Efficacy of Voice Therapy in Rehabilitation of Muscle Tension Dysphonia in Patients of Nonlaryngeal Head and Neck Cancer: A Sequelae of Chemoradiotherapy. Indian J Otolaryngol Head Neck Surg. 2023;75(4):3739-3749. doi:10.1007/s12070-023-04072-x | |
|  |  | 4. Anne B, Svea H, Benno P, Jorg W, Kartrin H. Effects of high-frequency speech therapy on speech-related quality of life and objective speech intelligibility of oral cancer patients. Journal of cranio-maxillo-facial surgery : official publication of the European Association for Cranio-Maxillo-Facial Surgery. 2021;49(11). doi:10.1016/j.jcms.2021.06.011 | |
|  |  | 5. Tuomi L, Björkner E, Finizia C. Voice outcome in patients treated for laryngeal cancer: efficacy of voice rehabilitation. J Voice. 2014;28(1):62-68. doi:10.1016/j.jvoice.2013.02.008 | |
|  |  | 6. Takahashi M, Yonezawa K, Morimoto K, Tanimoto H, Saito M, Otsuki N, Nibu K. Roles of speech therapists in the rehabilitation for patients with head and neck cancer. Japan J Head Neck Can. 2011;37(4):481-485. doi: 10.5981/jjhnc.37.481 | |
|  |  | 7. Lc O, Z K. A weekly speech and language therapy service for head and neck radiotherapy patients during treatment: maximizing accessibility and efficiency. The Journal of community and supportive oncology. 2015;13(7). doi:10.12788/jcso.0149 | |
|  |  | 8. Parsons A, Dewan K. Dysphagia and Dysphonia After Head and Neck Cancer. Oral Dis. Published online October 2024. doi:10.1111/odi.15152 | |
|  |  | 9. As B, S H, B P, J W, K H. Effects of high-frequency speech therapy on speech-related quality of life and objective speech intelligibility of oral cancer patients. Journal of cranio-maxillo-facial surgery : official publication of the European Association for Cranio-Maxillo-Facial Surgery. 2021;49(11). doi:10.1016/j.jcms.2021.06.011 | |
| Repeat published | 1 | 1.Angadi V. Investigating the efficacy of Vocal Function Exercises in improving vocal function in adults irradiated for laryngeal cancers: A three part dissertation. Theses and Dissertations--Rehabilitation Sciences. Published online January 1, 2016. doi:http://dx.doi.org/10.13023/ETD.2016.349 | |
| No relevant outcome indicators | 1 | Fan Q, Ying L, Zhou QQ, et al. Effect of Intermittent Respiratory Muscle Training on Rehabilitation of Patients with Head and Neck Cancer Undergoing Radiotherapy. Prog mod bio. 2024;24(7):1375-1379. doi:10.13241/j.cnki.pmb.2024.07.034 | |
