# Supplementary material for: Effectiveness of rehabilitation training on radiotherapy-related abnormalities of voice function in head and neck cancer patients: A systematic review and meta-analysis
Source: PLoS One. 2025 Mar 10;20(3):e0318577. doi: 10.1371/journal.pone.0318577 (PMC11892882; doi:10.1371/journal.pone.0318577)
Supplement: S6 Table — (DOCX) [file pone.0318577.s006.docx]

| **Study** | **Random sequence generation (selection bias)** | **Allocation concealment (selection bias)** | **Blinding of participants and personnel (performance bias)** | **Blinding of outcome assessment (detection bias)** | **Incomplete outcome data (attrition bias)** | **Selective reporting (reporting bias)** | **Other bias** |
| --- | --- | --- | --- | --- | --- | --- | --- |
| Angadi et al, 2020 | Low risk | Low risk | Low risk | Low risk | Low risk | Low risk | Low risk |
| Bergström et al, 2016 | Low risk | Low risk | Unclear risk | Unclear risk | Low risk | Low risk | Low risk |
| Eriksson et al, 2023 | Low risk | Low risk | High risk | Low risk | Low risk | Low risk | Low risk |
| Karlsson et al, 2015 | Low risk | Low risk | High risk | High risk | Low risk | Low risk | Low risk |
| Karlsson et al, 2017 | Low risk | Low risk | High risk | Low risk | Low risk | Low risk | Low risk |
| Karlsson et al, 2022 | Low risk | Low risk | High risk | Low risk | Low risk | Low risk | Low risk |
| Law et al, 2017 | Low risk | Low risk | Low risk | Unclear risk | Low risk | Low risk | Low risk |
| Liu et al, 2024 | Low risk | Low risk | Unclear risk | High risk | Low risk | Low risk | Low risk |
| Mantia et al, 2018 | Low risk | Low risk | Low risk | Low risk | Low risk | Low risk | Low risk |
| Millgard et al, 2020 | Low risk | Low risk | Unclear risk | Low risk | Low risk | Low risk | Low risk |
| Sreenivas et al, 2021 | Low risk | Low risk | Low risk | Low risk | Low risk | Low risk | Low risk |
| Tuomi et al, 2017 | Low risk | Low risk | High risk | Unclear risk | Low risk | Low risk | Low risk |
| Tuomi et al, 2014 | Low risk | Low risk | Unclear risk | Low risk | Low risk | Low risk | Low risk |

**S6 Table. Methodological quality of the trials**
