# Supplementary material for: Effectiveness of rehabilitation training on radiotherapy-related abnormalities of voice function in head and neck cancer patients: A systematic review and meta-analysis
Source: PLoS One. 2025 Mar 10;20(3):e0318577. doi: 10.1371/journal.pone.0318577 (PMC11892882; doi:10.1371/journal.pone.0318577)
Supplement: S7 Appendix — (DOCX) [file pone.0318577.s007.docx]

**Contents**

**1. S1 Fig. The forest plot of Jitter**

**2. S2 Fig. The forest plot of Shimmer**

**3. S3 Fig. The forest plot of NHR**

**4. S4 Fig. The forest plot of F0**

**5. S5 Fig. The forest plot of subgroup analysis for jitter**

**6. S6 Fig. The forest plot of subgroup analysis for shimmer**

**7. S7 Fig. The forest plots subjective evaluation of voice functions**

**8.** **S8 Fig. Sensitivity analysis**

**1.S1 Fig. The forest plot of Jitter**


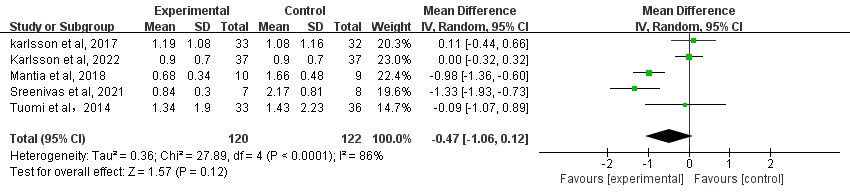


**2. S2 Fig. The forest plot of Shimmer**


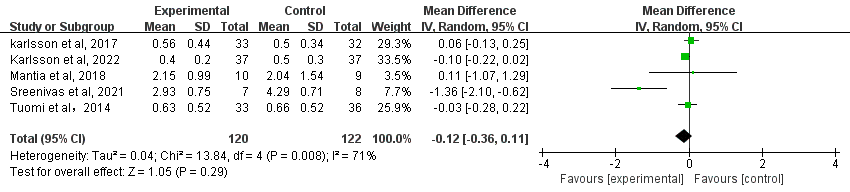


**3. S3 Fig. The forest plot of NHR**

**
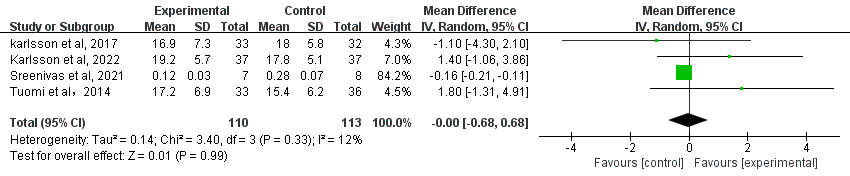
**

**4. S4 Fig. The forest plot of F0**

**
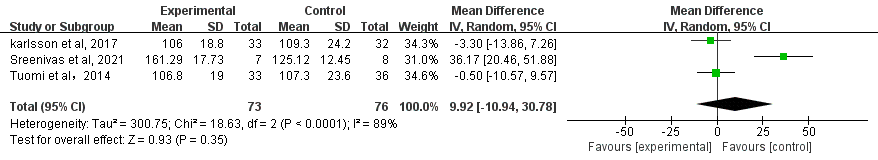
**

**5. S5 Fig. The forest plot of subgroup analysis for jitter**

**(a) Intervention frequency**

**
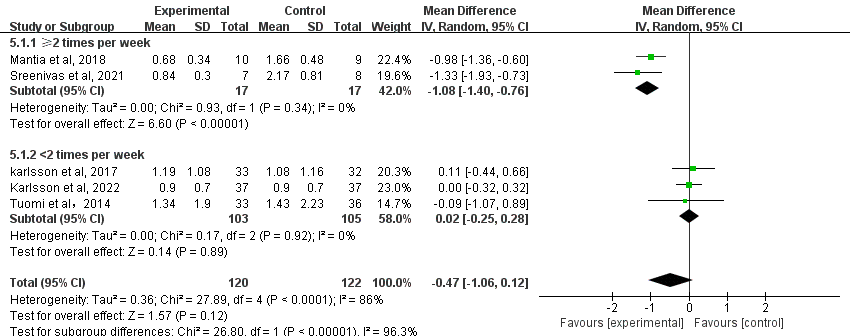
**

**(b) Intervention cycle**

**
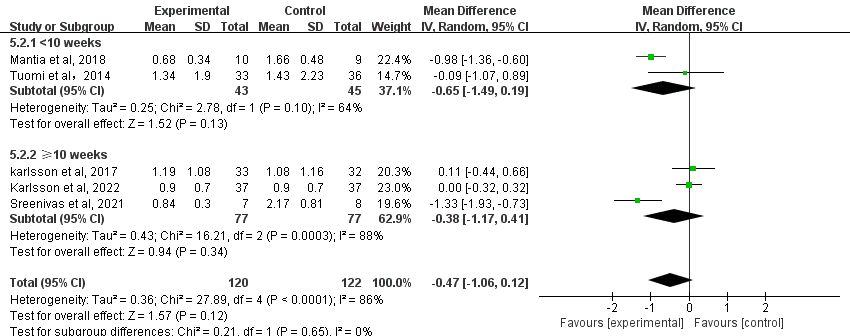
**

**(c) Follow-up Time**

**
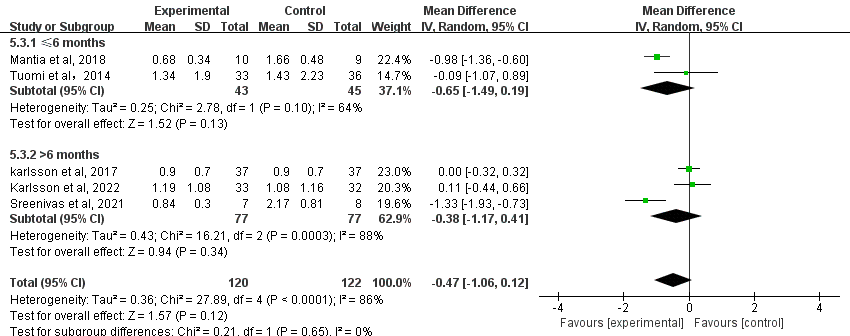
**

**6. S6 Fig. The forest plot of subgroup analysis for shimmer**

**(a) Intervention frequency**

**
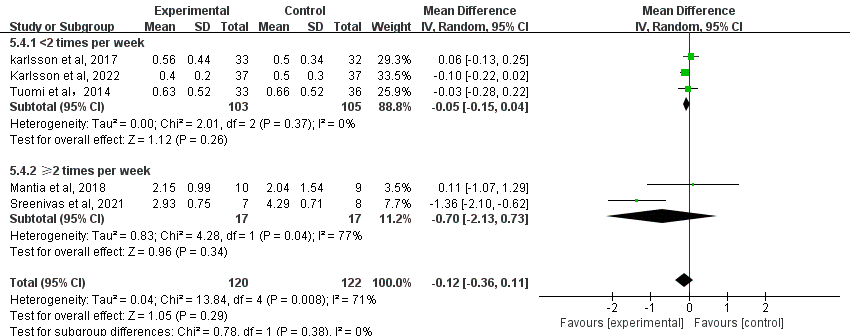
**

**(b) Intervention cycle**

**
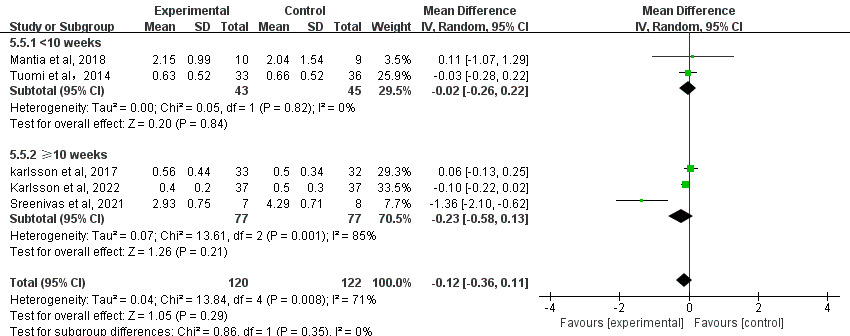
**

**(c) Follow-up Time**

**
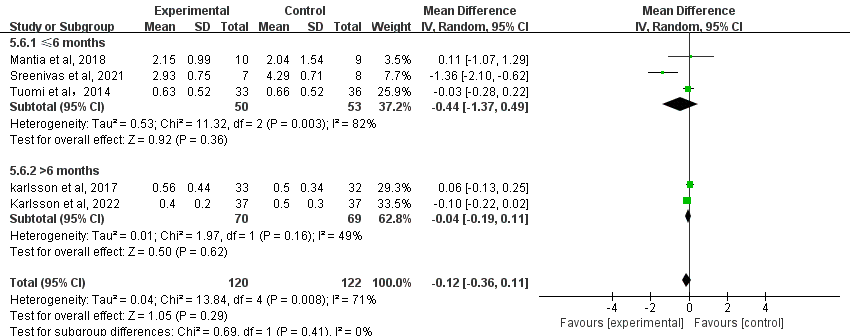
**

**7. S7 Fig. The forest plots subjective evaluation of voice functions**

**(a) Intervention cycle**

**
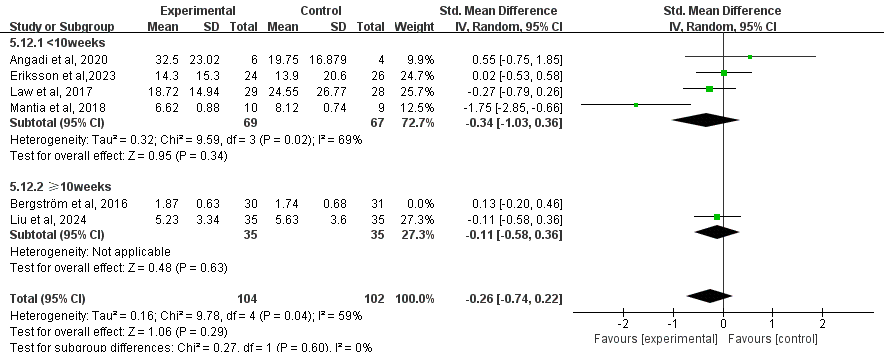
**

**(b) Follow-up Time**

**
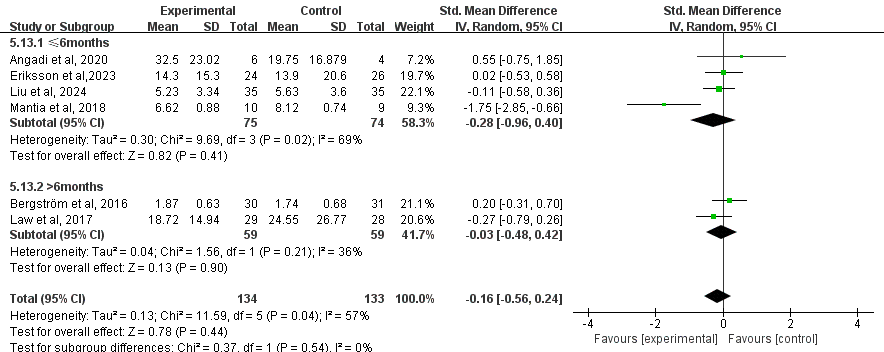
**

**8. S8 Fig. Sensitivity analysis**

**(a)** **Subjective evaluation of voice functions (b) MPT**

**
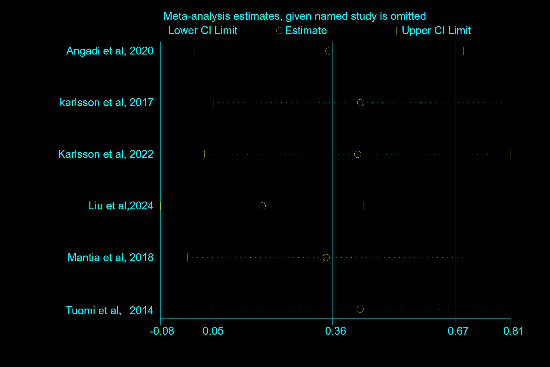

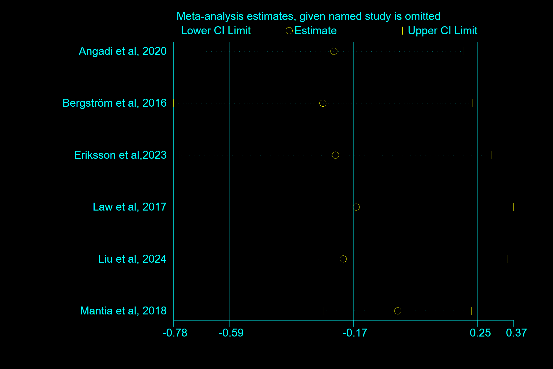
**

**(c) Jitter (d) Shimmer**

**
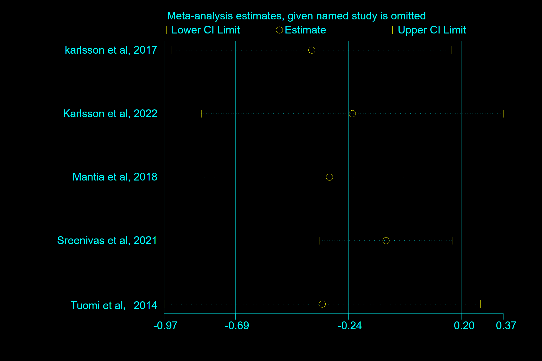

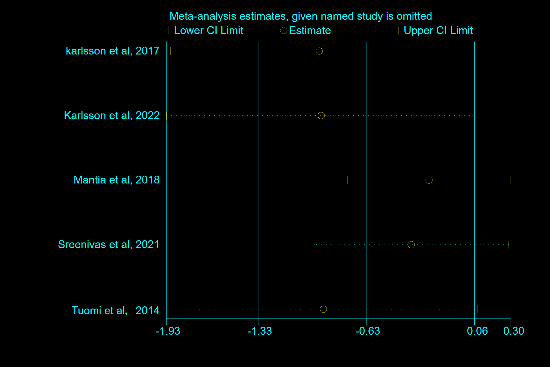
**

**(e) NHR (f) F0**

**
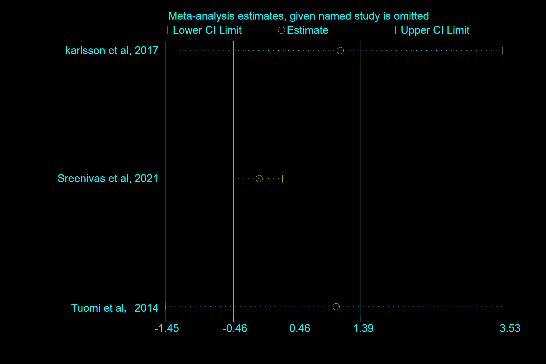

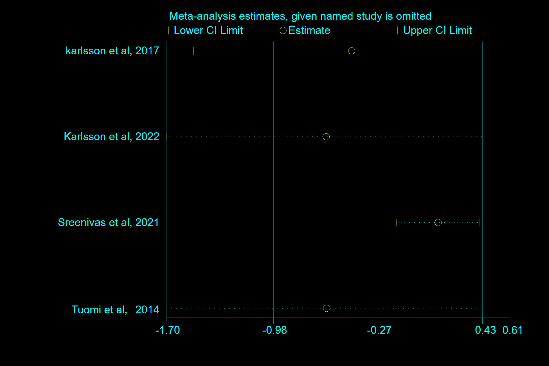
**

**(g) Social communication abilities (h) Quality of life**

**
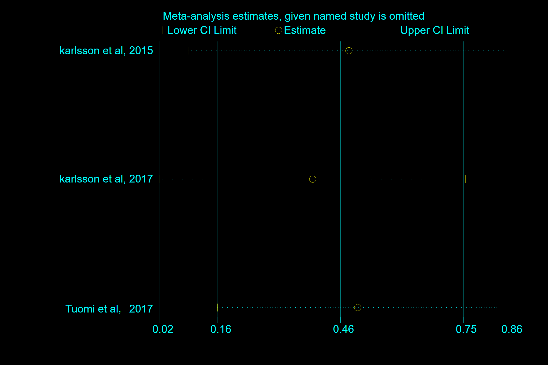

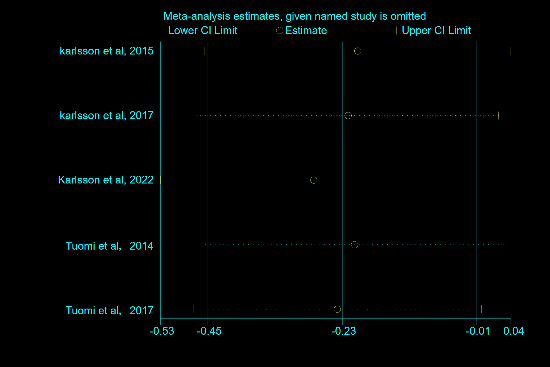
**
